# *Akkermansia muciniphila* ameliorates chronic stress-induced colorectal tumor growth by releasing outer membrane vesicles

**DOI:** 10.1080/19490976.2025.2555618

**Published:** 2025-09-08

**Authors:** Shunkang Jin, Yanjie Lu, Yanzhen Zuo, Qian Xu, Yanhui Hao, Hongyan Zuo, Zhilin Cui, Xinyu Zhang, Mengyun Wang, Hongwei Li, Shuai Wang, Yuhong Li

**Affiliations:** a Cancer Research Laboratory, Chengde Medical College, Chengde, Hebei, China; b Institute of Radiation Medicine, Academy of Military Medical Sciences, Academy of Military Sciences, Beijing, China; c Senior Department of Hepatology, The Fifth Medical Center of PLA General Hospital, Beijing, China

**Keywords:** Colorectal neoplasm, gut microbiota, chronic stress, *Akkermansia muciniphila*, outer membrane vesicles

## Abstract

Genetic predisposition and environmental factors, including psychological stress, play prominent roles in driving the development and progression of colorectal neoplasms. However, the mechanisms through which chronic stress drives the progression of colorectal neoplasm remain unclear. The gut microbiota is closely linked to chronic psychological stress (chronic stress) and colorectal neoplasms. Here, we found that chronic stress significantly promoted tumor growth in patients with colorectal cancer and mouse models of colitis-associated colorectal cancer, while concurrently reducing the abundance of *Akkermansia muciniphila* in fecal and tumor samples. Restoring the *A. muciniphila* abundance mitigated the tumor-promoting effects of chronic stress. Furthermore, we identified *A. muciniphila* outer membrane vesicles as key mediators of the protective effect of this microbe. In conclusion, *A. muciniphila* alleviates chronic stress-induced colorectal neoplasm growth by releasing outer membrane vesicles. These findings highlight a connection among chronic stress, the gut microbiota, and colorectal neoplasms, providing a theoretical foundation for therapeutic strategies aimed at managing tumor progression in patients with colorectal cancer experiencing chronic stress.

## SUMMARY

**Teaser** Enriching the gut microbiome with *A. muciniphila* can mitigate colorectal neoplasm growth.

## Introduction

Stress is a nonspecific systemic response of the body to internal and external environmental stimuli, including social and psychological factors. It is generally categorized as either acute or chronic. Chronic stress often exerts adverse effects on the body, involving the dysfunction of multiple systems, such as the nervous, immune, and digestive systems, which manifest as insomnia, appetite disturbances, and gastrointestinal dysfunction.^1,2^ Epidemiological studies have indicated that patients with cancer experience prolonged psychological stress compared with the general population.^3,4^ Eckerling et al. demonstrated that a cancer diagnosis can act as a persistent and intense stressor, leading to ongoing psychological distress in patients, characterized by anxiety, fear, and depression.^5–7^ In recent years, chronic stress has been increasingly recognized as a significant factor affecting the onset and progression of various cancers, with particularly pronounced effects on colorectal neoplasms. Trudel-Fitzgerald et al. demonstrated a negative correlation between chronic stress and survival rates in patients with colorectal cancer (CRC).^8^ However, the precise mechanisms through which chronic stress drives the progression of colorectal neoplasm remain unclear, underscoring the urgent need for further investigation.

The gut harbors a vast community of symbiotic microorganisms, including bacteria, fungi, viruses, archaea, and parasites.^9^ These gut microorganisms play a crucial role in maintaining intestinal homeostasis by metabolizing nutrients, regulating host immune function, and participating in host defense mechanisms.^10–13^ However, the gut microbial community is dynamic and susceptible to various factors, such as diet, medications, and psychological stress.^14^ Chronic stress alters the composition and metabolic functions of the gut microbiota in rats^15^ and is associated with reduced microbial richness and diversity.^16^ The disruption of the microbial balance in the gut is referred to as dysbiosis.^17^ Dysbiosis is strongly linked to several malignancies, including colorectal neoplasms, gastric cancer, hepatocellular carcinoma, breast cancer, pancreatic cancer, and lung cancer.^18–23^ Given the direct interaction between gut microbiota and colorectal neoplasm cells, dysbiosis is particularly significant in the progression of colorectal neoplasms.^9^ Wong et al. demonstrated that fecal transplantation from patients with CRC increases the tumor burden in germ-free colitis-associated colorectal cancer (CAC) mouse models compared with fecal transplantation from healthy individuals.^24^

Dysbiosis is characterized by a reduction in gut microbial diversity and alterations in the composition of resident species.^25^ Gut microorganisms can be categorized as harmful bacteria, commensal bacteria, and probiotics based on their effects on the host. The harmful gut bacterium *Fusobacterium nucleatum* induces a sharp reduction in N6-methyladenosine (m^6^A) expression in CRC cells by downregulating m^6^A methyltransferase 3, thereby promoting CRC invasion.^26^ Conversely, several probiotic strains exert inhibitory effects on colorectal neoplasms by releasing specific proteins and metabolites. For instance, *Streptococcus thermophilus* secretes β-galactosidase, which inactivates the oncogenic Hippo signaling pathway, thereby suppressing CAC development.^27^ Indole-3-lactic acid produced by *Lactobacillus gallinarum* can prevent CAC tumor formation in mice.^28^

Existing evidence alludes to an intrinsic sequential relationship linking “chronic stress – commensal bacteria imbalance – colorectal neoplasm.” Thus, investigating alterations in the gut microbiota under chronic stress could enhance our understanding of the mechanisms by which chronic stress affects the progression of colorectal neoplasms. Moreover, identifying the key commensal bacteria involved in this process could lead to novel therapeutic strategies for mitigating the progression of chronic stress-induced colorectal neoplasms. Thus, in the present study, we investigated the effect of chronic stress on colorectal neoplasm progression. Our results indicate that chronic stress promotes colorectal neoplasm progression by reducing the abundance of *A. muciniphila* in the gut and within colorectal tumors, thereby weakening the tumor-suppressive effects mediated by *A. muciniphila* outer membrane vesicles (OMVs). These findings provide novel insights into the mechanisms underlying chronic stress-induced colorectal neoplasm progression and offer a theoretical foundation and scientific basis for the development of related therapeutic strategies.

## Materials and methods

### Patient recruitment

The clinical protocol was approved by the Ethics Committee of Chengde Medical College (Approval No.: 202215) and the study was conducted in accordance with the ethical principles outlined in the Declaration of Helsinki. All participants were recruited from the Affiliated Hospital of Chengde Medical College between May 2022 and September 2023. Before the intervention, all eligible participants provided informed consent.

#### Chronic stress assessment in patients

Anxiety and depression in patients with CRC were assessed using Zung’s Self-Rating Anxiety Scale (SAS) and Self-Rating Depression Scale (SDS).^29^ The inclusion criteria were: (1) histologically diagnosed CRC; (2) aged between 18 and 80 years; (3) not currently taking antidepressants or antipsychotic medications; (4) ability to communicate effectively with healthcare providers; and (5) willing to voluntarily participate in questionnaire assessment. The exclusion criteria were: (1) severe mental illness or cognitive impairment; (2) hearing or speech disabilities preventing effective communication; (3) an expected survival time of less than 3 months; and (4) unwilling to participate in the study. SAS and SDS contain 20 items, with scores ranging from 20 to 80. Anxiety is defined as an SAS score ≥50 and depression is defined as an SDS score ≥53. Patients exhibiting anxiety or depression were categorized as experiencing chronic stress.

#### Tumor tissue collection

The tumor tissue collection procedure followed a protocol previously reported by our research group.^30^ The inclusion criteria for tumor tissue collection were: (1) histologically diagnosed CRC; (2) no prior history of colorectal surgery; (3) no specific dietary habits; (4) no use of antibiotics, corticosteroids, or probiotics in the month preceding surgery; (5) no familial adenomatous polyposis or hereditary non-polyposis CRC; and (6) voluntary participation with written informed consent. The exclusion criteria were: (1) adjuvant radiotherapy or chemotherapy; (2) pregnant or lactating; (3) a previous cancer diagnosis; and (4) unwillingness to participate in the study.

### Animal experiments

Animal experiments were approved by the Ethics Committee of the Experimental Animal Center of the Academy of Military Medical Sciences (Approval No.: IACUC-DWZX-2022–854). All animal procedures were performed according to the National Institutes of Health Guide for the Care and Use of Laboratory Animals. The experimental mice were purchased from Beijing Vital River Laboratory Animal Technology Co., Ltd. (Animal Quality Certificate: SCXK (Jing) 2021–0006) and housed at the Experimental Animal Center of the Academy of Military Medical Sciences. The environment was maintained at 22–25°C, with a relative humidity of 40%–60% and 12-h light/dark cycle.

#### CAC model

Eight-week-old male C57BL/6J mice (20–22 g) were used.^31^ The mice underwent a 7-day acclimation period before the experiment. On day 0, they received an intraperitoneal injection of 10 mg/kg azoxymethane (AOM) (catalog no. A5486, Sigma Aldrich). One week later, they were administered 2% dextran sulfate sodium (DSS) solution (catalog no. 0216011090, MP Biomedicals) ad libitum for 7 days, followed by ddH_2_O for 14 days. This constituted one DSS cycle. Three DSS cycles were performed.

#### Chronic stress model

The chronic unpredictable mild stress (CUMS) paradigm was used as a chronic stress model in mice following the procedure described by Song et al.^32^ Briefly, mice were subjected to various stressors in a prolonged, mild, and unpredictable manner for 14 days to induce chronic stress. The applied stressors included: (1) placement in a cage without bedding (6 h); (2) placement in a cage with a wet floor (6 h); (3) placement in a dirty cage with an aversive odor (6 h); (4) wet bedding (6 h); (5) cage tilting at a 45° angle (24 h); (6) noise exposure (15 min); (7) foot shock (200 μA, 20 random shocks over 30 min, each lasting 1 s); (8) ice-water swimming (15°C, 5 min); (9) chronic restraint (6 h in a 50 mL centrifuge tube); and (10) inversion of the 12:12 h light/dark cycle. To prevent adaptation, the stressors were applied in a pseudo-randomized order to ensure unpredictability. In addition, no stressors were applied within 12 h before behavioral testing to minimize behavioral bias. During the entire experimental period, all mice were provided ad libitum access to food and water.

#### Fecal microbiota transplantation (FMT) model

The procedure was performed following a previously published protocol.^33^ A broad-spectrum antibiotic solution (Abx) was prepared by dissolving 1 g/L ampicillin (catalog no. A9518, Sigma-Aldrich), 1 g/L metronidazole (catalog no. M3761, Sigma-Aldrich), 0.5 g/L neomycin (catalog no. N1876, Sigma-Aldrich), and 0.5 g/L vancomycin (catalog no. 94747, Sigma-Aldrich) in distilled water. Mice were administered this Abx once daily via oral gavage at a dose of 20 μL per gram of body weight for 7 consecutive days to deplete the gut microbiota. During this period, mice were allowed only deionized water (ddH_2_O) to avoid any interference with the antibiotic treatment.

Fresh feces were collected from donor mice and placed in pre-cooled sterile tubes. Sterile phosphate-buffered saline (PBS) was added at a concentration of 100 mg/mL and the mixture was thoroughly homogenized. The fecal suspension was filtered through a mesh to remove solid particles. After centrifugation at 500 × *g* for 3 min at 4°C, the supernatant was collected. Following a second centrifugation, the resulting fecal microbiota suspension was stored at −80°C until use. Recipient mice were administered 200 μL of the fecal microbiota suspension once daily via oral gavage for 14 consecutive days following the 7-day Abx treatment to restore the gut microbiota.

### Behavioral assessments

Three days before behavioral assessments, the mice were acclimated to the behavioral testing room for 30 min each day and gently handled to reduce stress. All behavioral tests were conducted the day after the experimental intervention was completed. To minimize the variability caused by fatigue and transport-related stress, each mouse underwent only one behavioral test per day. The mice were allowed to adapt to the test environment for 1 h before each test. During the testing phase, the surroundings were kept quiet to prevent disturbance. After each mouse completed the test, feces and urine residues were removed and the inner walls and floor of the apparatus were wiped with 75% ethanol to eliminate odor interference before testing the next mouse. This standard cleaning procedure was strictly followed for all behavioral assessments.

#### Open field test (OFT)

The OFT was used to assess anxiety levels in mice.^34^ During testing, each mouse was placed individually in the center of an open-field arena (40 × 40 × 35 cm), where the central area was defined as the inner 20 × 20 cm region and the remaining space was defined as the peripheral area. Each mouse was placed in the center with its back facing the experimenter and allowed to explore freely for 5 min. The movement trajectory of each mouse was recorded using an automated activity-monitoring system (ANY-maze software version 6.32, Stoelting, USA).

#### Tail suspension test (TST)

The TST was used to assess depression-like behavior in mice.^35^ During the test, each mouse was suspended by the tail using medical adhesive tape securely affixed to the top of a testing chamber (49 × 29 × 45 cm). The mouse was allowed to struggle freely for 6 min and its movement trajectory was recorded using an automated activity monitoring system (DigBehv Animal Behavior Video Analysis System, Version DigBehv 4.0; Shanghai Jiliang Software Technology Co., Ltd, China). Only the final 4 min of the 6-minute session were analyzed to evaluate the duration of immobility.

### A. muciniphila *cultivation and administration*

#### *Cultivation of* A. muciniphila

*A. muciniphila* (ATCC BAA-835) was purchased from Mingzhou Bio Co., Ltd. (Ningbo, China).^36^ After thawing, *A. muciniphila* was inoculated into Chopped Meat Carbohydrate Broth (CN) (catalog no. KDM150, Mingzhoubio) and cultured under anaerobic conditions at 37°C for 72 h in an anaerobic chamber (Bactron EZ-2, Shellab, USA). *A. muciniphila* was subsequently stored in anaerobic gas packs at 4°C for transport to our laboratory.

#### *Administration of A.* muciniphila

Mice received 200 μL of *A. muciniphila* suspension (1 × 10^9^ CFU) via oral gavage daily for 14 consecutive days.^37^ To ensure the viability of *A. muciniphila* during administration, bacterial suspensions were delivered in 4-mL batches daily.

### Experiments on A. muciniphila OMVs

#### *Extraction and characterization of* A. muciniphila *OMVs*

The extraction and characterization of *A. muciniphila* OMVs were performed following a previously described protocol.^38^ *A. muciniphila* supernatant was obtained from Mingzhoubio Co., Ltd. (Ningbo, China). *A. muciniphila* was cultured under anaerobic conditions at 37°C for 72 h. The culture was centrifuged at 4°C, 5000 × *g* for 10 min to obtain the supernatant. The supernatant was then centrifuged at 4°C 15,000 × *g* for 20 min and filtered through a 0.45-μm filter to remove bacterial particles and other impurities. The filtered solution was further centrifuged at 4°C, 170,000 × *g* for 80 min to pellet *A. muciniphila* OMVs. The pellet was resuspended in sterile PBS (catalog no. G4250, Servicebio) and centrifuged again at 4°C, 170,000 × *g* for 60 min to purify the *A. muciniphila* OMVs. After ultracentrifugation, the pellet was resuspended in sterile PBS (catalog no. G4250, Servicebio) under aseptic conditions and filtered through a 0.45-μm filter to obtain sterile *A. muciniphila* OMV solution. The purified OMVs were stored at −80°C for further use. Nanoparticle tracking analysis (NTA) and transmission electron microscopy (TEM) were used to characterize the extracted *A. muciniphila* OMVs.

#### Administration of A. muciniphila OMVs

Mice received 100 μL of *A. muciniphila* OMV suspension (containing 20 μg of protein) via oral gavage daily for 14 consecutive days.^38^

#### Stability of A. muciniphila OMVs

The stability of *A. muciniphila* OMVs was assessed by incubating them in simulated gastric fluid (SGF) (catalog no. SL6600A, Coolaber) and simulated intestinal fluid (SIF) (catalog no. SL66102, Coolaber) at 37°C for 2 and 4 h, respectively. NTA was used to assess the concentration and size of *A. muciniphila* OMVs after incubation.

#### Protein enrichment of A. muciniphila OMVs

To analyze protein enrichment in *A. muciniphila* OMVs, samples were heated in a metal bath at 100°C for 10 min, followed by SDS-PAGE for 60 min. The gel was stained with Coomassie Brilliant Blue (CBB) stain (catalog no. P0017, Beyotime) for 30 min, followed by destaining overnight in a solution of acetic acid: ethanol: water (1:3:6). Label-free quantitative mass spectrometry (LC-MS/MS) was performed on three independently prepared batches of *A. muciniphila* OMVs to identify and quantify enriched proteins.

### Serological testing

We used a radioimmunoassay (RIA) to measure epinephrine (EPI) and norepinephrine (NE) levels in mouse blood samples. After completing the behavioral assessments, the mice were euthanized and blood samples were collected via intraperitoneal extraction. The collected blood was centrifuged at 4°C, 3500 rpm for 10 min to obtain serum. The serum was transferred to new Eppendorf tubes and stored at −80°C for subsequent RIA analysis.

#### RIA protocol

(i) 200 μL of buffer solution was added to the nonspecific binding (NSB) tube, 100 μL of buffer solution was added to the zero standard (S0) tube, 100 μL of serum sample was added to the respective sample tubes, and 100 μL of primary antibody was added to all tubes; (ii) 100 μL of radiolabeled EPI and NE marker was added to all tubes, mixed thoroughly, and incubated overnight at 4°C; (iii) 500 μL of secondary antibody separation reagent was added to all tubes, mixed thoroughly, and centrifuged at 4°C for 25 min; and (iv) the supernatant was discarded and radioactivity (counts per minute, CPM) in the precipitate of each tube was measured (T represents the total radioactivity, B is the standard sample, and B0 is the radioactivity count of the standard sample at time zero). The NSB and S0 binding percentages were calculated as B/T, whereas the binding percentages of the standard and test samples were calculated as B/B0. A standard curve was plotted on a semi-logarithmic graph and sample values were determined accordingly.

### Histopathological evaluation and immunohistochemistry (IHC)

Following euthanasia, the colon specimens were immediately excised from the mice and fixed in 10% formalin solution for 3 days. The colon tissue was subsequently embedded in paraffin and sectioned in a mucosa-to-serosa orientation.

#### Histopathological evaluation

Tissue sections were stained with hematoxylin (catalog no. G1080, Solarbio) and eosin (catalog no. G1100, Solarbio) for histopathological examination. Histopathological scoring was performed according to a previously published consensus report.^33^ The scoring criteria were defined as: scores of 0, 1, 2, 3, and 4 represented normal, mild (inflammation or epithelial loss), moderate (elongation of crypts with crowded hyperchromatic epithelium, loss of crypt branching; slightly enlarged, crowded, and hyperchromatic nuclei; marginally reduced intracellular mucin, marked (crypts 2–3 times thicker than normal, hyperchromatic epithelium, reduced goblet cells, scattered arborization), and severe (crypts 2–3 times thicker than normal, marked hyperchromasia, few or no goblet cells, high mitotic index, frequent arborization), respectively. The slides were reviewed and scored by several experienced clinical pathologists. To minimize subjective bias, the sample group identities were concealed during the evaluation process.

#### IHC

After dewaxing and rehydrating the tissue sections, antigen retrieval was performed using a citrate buffer (catalog no. ZLI-9064, ZSGB-bio) for 15 min (5 min at high heat, followed by 10 min at medium-to-low heat). Following PBS washes, the sections were incubated with an endogenous peroxidase blocker solution (catalog no. PV-9001, ZSGB-Bio) for 30 min. After another PBS wash, the sections were incubated overnight at 4°C with an anti-Ki67 primary antibody solution (1:400, catalog no. 12202S, Cell Signaling Technology). After washing with PBS, the sections were incubated with a secondary antibody solution (catalog no. PV-9001; ZSGB-Bio) for 30 min. After another PBS wash, DAB solution (1:200, catalog no. ZLI-9017, ZSGB-Bio) was applied for 60 s for color development. After terminating the color reaction, the sections were counterstained with hematoxylin staining solution (catalog no. G1080, Solarbio). Finally, 3–5 random microscopic fields were selected from each tissue section for quantitative analysis using ImageJ.

### 16S rRNA gene sequencing

Fresh intestinal contents were collected for 16S rRNA gene sequencing. Microbial DNA was extracted from fecal samples using a standardized protocol. The V4 hypervariable region of the 16S rRNA gene was amplified and sequenced using high-throughput platforms. Raw sequencing reads were quality-filtered and denoised to generate high-quality consensus sequences. Operational taxonomic units were clustered at a 97% sequence similarity threshold using USEARCH (v10.0) and taxonomic annotation was performed against the Silva reference database using a Naive Bayes classifier.

To assess differences in microbial composition between groups, we performed differential abundance analysis using Metastats, a non-parametric, permutation-based statistical test specifically designed for microbiome data that do not require the assumption of normality. To correct for multiple comparisons, false discovery rate (FDR) adjustment was applied and significantly altered taxa were identified based on adjusted *P*-values < 0.05.

β-diversity analysis was performed based on Bray – Curtis distance metrics and visualized using principal coordinates analysis (PCoA). To assess the statistical significance of differences in community structure between groups, we applied PERMANOVA (Adonis) using 999 permutations.

### Cancer cachexia

Body weight, stool consistency, and intestinal bleeding of the experimental mice were recorded following exposure to stress factors and monitored until the end of the experiment. Ethical guidelines for animal welfare were strictly followed and mice exhibiting more than 18% body weight loss combined with lethargy were euthanized.

#### Body weight change rate

The body weight of the experimental mice was recorded one day before stress factor treatment and defined as the initial body weight. The rate of body weight change was calculated using the formula: (current body weight/initial body weight) × 100.

#### Disease activity index (DAI)

The DAI system is based on three components: body weight loss ratio, stool consistency, and intestinal condition. The DAI was scored based on a previously published consensus report (Table 1).^39^ The body weight loss ratio was calculated using the formula: ((Current body weight − Initial body weight)/Initial body weight) × 100.

**Table 1.** Disease activity index.

Weight loss	Feces consistency	Intestinal bleeding	Score
None	Normal	Normal	0
0% − 5%	Soft but formed	Blood traces in stool visible	1
5% − 10%	Soft and unformed	Blood traces in stool	2
10% − 18%	Very soft and wet	Archorrhagia	3
> 18%	Diarrhea	Rectocele	4

The overall score is the sum of the three individual scores divided by three, ranging from 0 (no change) to 4 (severe disease activity).

### Quantification of A. muciniphila abundance

To ensure the accuracy of *A. muciniphila* abundance quantification, fecal samples were continuously collected for 3 days, starting 72 h after the completion of the 14-day oral gavage of *A. muciniphila*. Total DNA was extracted from the fecal samples using a Fecal Genomic DNA Extraction Kit (catalog no. DP328–02, Tiangen). Quantification of *A. muciniphila* abundance in the fecal samples was performed using quantitative polymerase-chain reaction (qPCR). The qPCR consisted of: 2 µL of template DNA, 10 µL of SYBR qPCR Master Mix (catalog no. Q712–03, Vazyme), 0.4 µL of forward primer (10 µM), 0.4 µL of reverse primer (10 µM), and 7.2 µL of ddH2O.^40^ Ct values were recorded and relative gene expression levels were calculated using the 2^−ΔΔCt^ method. Primer sequences: 1. *A. muciniphila* forward primer: 5′-CAGCACGTGAAG-TGGGAC-3′; 2. *A. muciniphila* reverse primer: 5′-CCTTGCGGTTGGCTTCAGAT-3′; 3. Fecal internal control forward primer: 5′-CGGCAACGAGCGCAACCC-3′; and 4. Fecal internal control reverse primer: 5′-CCATTGTAGCACGTGTGTAGCC-3′. All primers were synthesized by Sangon Biotech Co., Ltd. (Shanghai, China).

*A. muciniphila* abundance in tumor tissues was measured using an absolute quantification PCR method. A standard curve was generated from a series of known DNA concentrations to convert Ct values into bacterial copy numbers. To ensure sample comparability, all tumor tissues were consistently sampled from the central region of each tumor and equal tissue quantities were used for DNA extraction. Furthermore, equal volumes of extracted DNA (1 µL) were used for each qPCR.

### Cell culture experiments

The HCT116 and SW620 cell lines were purchased from Procell Life Science & Technology Co., Ltd. (Wuhan, China). HCT116 cells were cultured in RPMI-1640 medium and SW620 cells were cultured in Dulbecco’s modified Eagle’s medium. Both media were supplemented with 10% fetal bovine serum (catalog no. 10099-141C; Gibco) and 1% penicillin-streptomycin (catalog no. 15140–122, Gibco). All cells were maintained at 37°C in a humidified incubator with 5% CO_2_.

#### Cell proliferation assay

Cell viability was assessed using a CCK-8 Kit (catalog no. 40203ES88, Yeasen), following the manufacturer’s instructions. Briefly, cells were seeded into 96-well plates with six replicates per group. After incubation with treatment factors for 2 days, 100 µL of the CCK-8 working solution (1:9) was added to each well. After a 2-h incubation, the absorbance was measured at 450 nm using a microplate reader.

#### Colony formation assay

On day 0, the cells were seeded into 6-well plates. On day 1, after cell adherence, 20 µg of *A. muciniphila* OMVs was added and cells were incubated for 2 days. On day 4, the culture medium was replaced with the standard growth medium and incubation was continued for another 11 days. On day 15, colonies were fixed with methanol for 10 min and stained with 0.1% crystal violet at room temperature for 15 min. Colony numbers were quantified using ImageJ.

#### Uptake of A. muciniphila OMVs

To assess the uptake of *A. muciniphila* OMVs,^41^ OMVs were labeled with Dil (catalog no. D3911, Thermo Fisher Scientific) for 1 h in the dark. Unbound dye was removed via ultracentrifugation at 200,000 × *g* for 1.5 h. The labeled OMVs were cocultured with the cell lines for 10 h. After incubation, the cells were washed three times with PBS. The actin cytoskeleton was stained with Actin Green 488 Ready Probe (catalog no. R37110, Thermo Fisher Scientific) in the dark for 30 min. Cell nuclei were stained and fixed using VECTASHIELD Antifade Mounting Medium (catalog no. H-1200, Vector). The uptake of *A. muciniphila* OMVs by CRC cells was observed using laser-scanning confocal microscopy.

### Quantification and statistical analysis

All experimental data are presented as mean ± standard error of the mean (SEM). Statistical analyses were performed using SPSS (version 27.0) and graphical representations were generated using GraphPad Prism (version 8.0). To minimize the effect of potential confounding factors when assessing variables associated with tumor volume in patients with CRC, we performed a Type II analysis of variance (ANOVA), with tumor volume as the dependent variable and stress status, sex, and age as independent variables. Data normality was assessed using the Shapiro–Wilk test. For normally distributed data, comparisons between two groups were conducted using an independent samples *t*-test, whereas comparisons among three or more groups were analyzed using one-way ANOVA. For non-normally distributed data, the non-parametric Mann – Whitney *U* test was applied. Microbiome data were analyzed using Metastats, a permutation-based non-parametric method tailored for microbiome datasets, with FDR correction applied to account for multiple comparisons. A *P*-value <0.05 was considered statistically significant.

## Results

### Chronic stress promotes colorectal neoplasm growth

**Figure 1.**
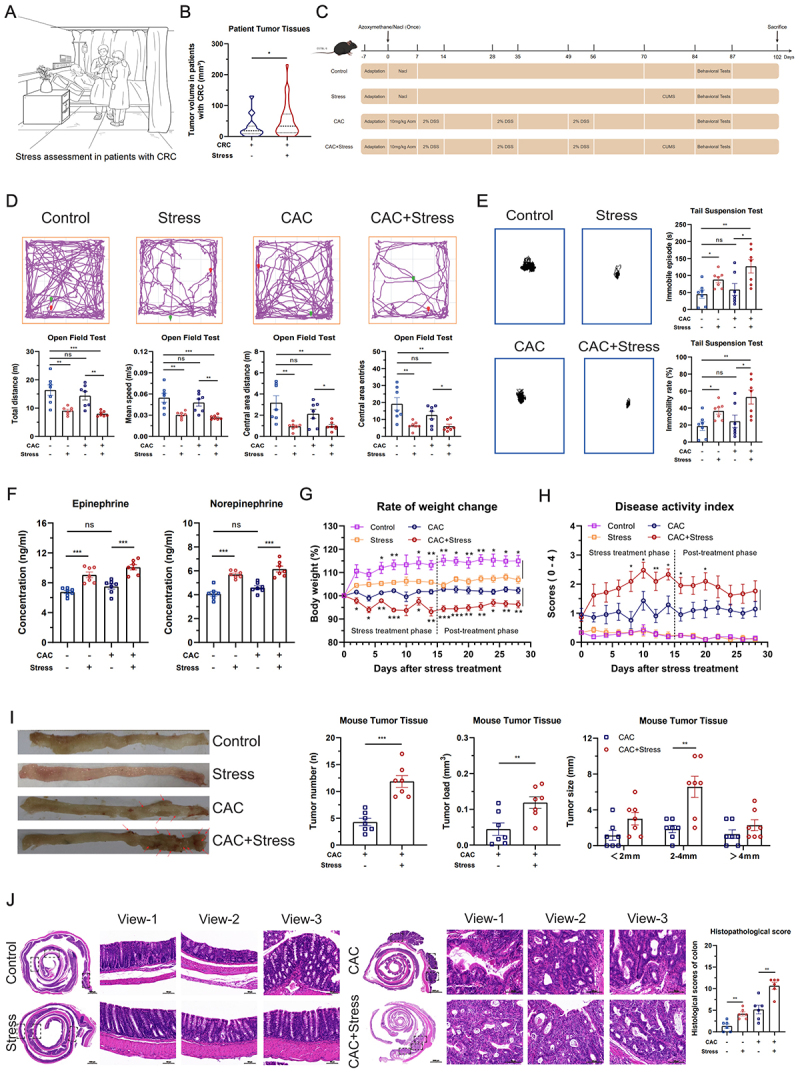
Chronic stress promotes the growth of colorectal neoplasms. (A) Schematic diagram of chronic stress assessment in patients with colorectal cancer (CRC). Patients exhibiting either anxiety (Self-Rating anxiety Scale score ≥ 50) or depression (Self-Rating depression Scale score ≥ 53) were categorized as experiencing chronic stress. (B) Tumor volume in stressed (*n* = 32) and non-stressed (*n* = 33) patients with CRC (total *n* = 65). (C) Establishment of the chronic stress-induced colitis-associated colorectal cancer (CAC) mouse model. Behavioral performance of experimental mice in (D) Open field test and (E) tail suspension test (*n* = 7). (F) Serum levels of epinephrine and norepinephrine (*n =* 7). (G) Rate of body weight change in experimental mice (*n* = 7). (H) Disease activity index of experimental mice (*n* = 7). (I) Quantitative analysis of tumor burden in CAC mice under chronic stress. Tumor number (left), tumor load (middle), and tumor size distribution (right) were assessed in the CAC and CAC+Stress groups (*n* = 7). Representative images of tumors from each group are displayed to the left of the bar graphs. Red arrows indicate individual tumors. (J) Histopathological analysis of tumor tissues under chronic stress conditions (*n* = 6). Scale bar = 100 μm. Data are presented as means ± SEM. Statistical significance was assessed using the Mann–Whitney *U* test, one-way ANOVA, or an independent samples *t*-test, as appropriate. *p-*values: ^ns^ *p* > 0.05; *p < 0.05; **p < 0.01; ***p < 0.001.

To evaluate the impact of chronic stress on tumor growth in patients with CRC, we first used the SAS and SDS to classify patients according to the presence or absence of chronic stress (Figure 1(A)). Subsequently, clinical variables, such as sex, age, and stress status, were systematically collected and tabulated. Multivariate analysis revealed that stress status was the only independent factor significantly associated with tumor volume (*p* = 0.022), whereas age and sex did not show significant associations (*p* = 0.898 and *p* = 0.144, respectively). Consistently, the results of the Mann – Whitney U test corroborated those of the multivariate analysis, revealing significantly larger tumor volumes in stressed patients with CRC than in non-stressed counterparts (Figure 1(B)). These findings collectively suggest that chronic stress is a key contributing factor to tumor progression in patients with CRC.

The AOM/DSS-induced CAC model is widely used in colorectal neoplasm research owing to its strong histopathological and molecular similarity to human CRC.^42^ This model induces colorectal tumors through repeated epithelial injury,^43^ which inevitably causes persistent (chronic) pain. As chronic pain is a well-recognized stressor capable of eliciting physiological stress responses,^44,45^ we first sought to determine whether pain induced by CAC modeling could confound the interpretation of chronic stress-related outcomes. To address this, male and female C57BL/6J mice were exposed to various combinations of AOM and DSS (Figure S1(A)). Behavioral assessments showed no significant differences in anxiety- and depression-like behaviors among treatment groups, indicating that the CAC modeling process itself did not elicit a chronic stress phenotype (Figures S1(B–K) and S2(A–J)).

To evaluate the effect of chronic stress on CAC progression in mice, we exposed CAC mice to CUMS for 14 days (Figure 1(C)), establishing a chronic stress-induced CAC mouse model. To avoid potential hormonal interference and ensure consistency across experimental groups, we used only male mice in subsequent experiments as endogenous estrogen in female mice has been reported to affect CRC development.^46^ Anxiety- and depression-like behaviors were assessed using the OFT and TST. Compared with non-stressed CAC mice, stressed CAC mice exhibited a significantly reduced total distance traveled, fewer entries into the center area, and decreased movement in the central region, along with a reduction in the mean movement speed in the OFT (Figure 1(D)). Additionally, stressed CAC mice displayed a significant increase in immobility time and percentage in the TST (Figure 1(E)). To confirm the chronic stress state in CAC mice, we measured the adrenal gland volume and serum levels of EPI and NE. Stressed CAC mice had significantly enlarged adrenal glands (Figure S3A) and elevated serum EPI and NE levels compared with non-stressed CAC mice (Figure 1(F)). During this period, we monitored the changes in body weight and DAI to investigate the effect of chronic stress on cancer cachexia in CAC mice. Stressed CAC mice exhibited significantly higher weight loss (Figure 1(G)) and DAI (Figure 1(H)) than their non-stressed counterparts.

To assess the effect of chronic stress on tumor progression, we performed gross colon tissue observation, IHC, and histopathological scoring. Gross observation of the colon tissue revealed that intestinal tumors were present only in mice subjected to the AOM/DSS treatment, specifically those in the CAC and CAC+Stress groups. Chronic stress exacerbated tumor growth, as indicated by more tumors, greater tumor load, and higher proportion of tumors measuring 2–4 mm in the CAC+Stress group than in the CAC group (Figure 1(H)). IHC revealed that chronic stress significantly increased the number of Ki67-positive proliferative cells in tumor tissues (Figure S3B,C). Histopathological scoring confirmed that chronic stress exacerbated the malignant progression in CAC mice (Figure 1(J)).

### Chronic stress-induced gut dysbiosis promotes the growth of colorectal neoplasms

Previous mechanistic studies investigating the role of chronic stress in cancer progression have primarily focused on the hypothalamic–pituitary–adrenal (HPA) axis and sympathetic nervous system.^47,48^ For instance, Zhou et al. demonstrated that adrenaline produced by the HPA axis promotes CRC stemness through the CEBPB-TRIM2-P53 pathway.^49^ However, Cao et al. did not report any significant difference in adrenaline and cortisol levels between the intestinal regions of stressed and non-stressed mice,^33^ suggesting the existence of additional contributing factors that remain unknown. In our investigation on the mechanisms by which chronic stress regulates the progression of colorectal neoplasms, we observed distinct differences in fecal consistency between the CAC and CAC+Stress groups (Fig. S4A). Given the critical role of the gut microbiota in tumor progression and emotional regulation,^9,50–52^ we hypothesized that the gut microbiota may be involved in the growth of chronic stress-mediated colorectal neoplasms.

**Figure 2.**
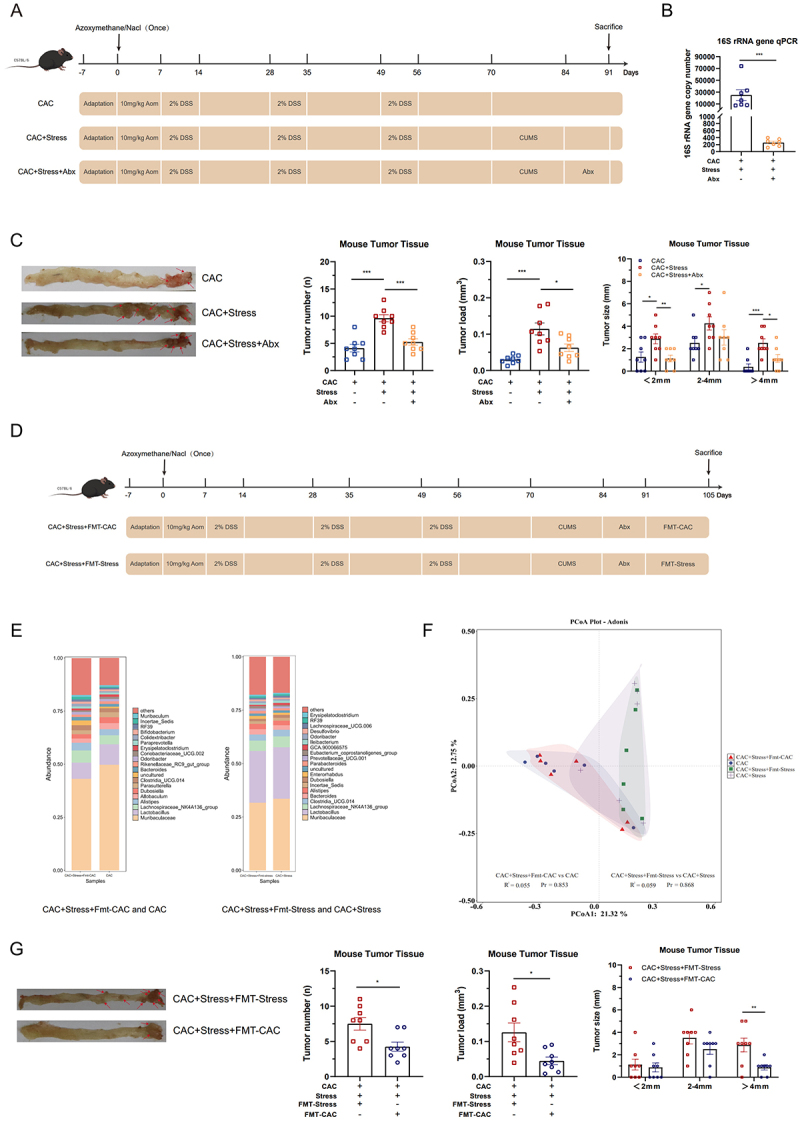
Chronic stress-induced gut dysbiosis promotes the growth of colorectal neoplasms. (A) Schematic diagram of the antibiotic (Abx) model. (B) Total bacterial load was quantified using the 16S rRNA gene quantitative polymerase chain reaction (qPCR; *n* = 7). (C) Quantitative analysis of tumor burden in colitis-associated colorectal cancer (Cac)+stress mice under Abx treatment. Tumor number (left), tumor load (middle), and tumor size distribution (right) were assessed in the CAC+Stress and CAC+Stress+Abx groups (*n* = 8). Representative images of tumors from each group are displayed to the left of the bar graphs. Red arrows indicate individual tumors. (D) Schematic diagram of fecal microbiota transplantation (FMT) model. (E and F) genus-level taxonomic profiling and principal coordinate analysis (PCoA) of β-diversity were performed on fecal samples from donor and recipient mice (*n* = 6). (G) Quantitative analysis of tumor burden in CAC+Stress mice under FMT. Tumor number (left), tumor load (middle), and tumor size distribution (right) were assessed in the CAC+Stress+FMT-CAC and CAC+Stress+FMT-Stress groups (*n* = 8). Representative images of tumors from each group are displayed to the left of the bar graphs. Red arrows indicate individual tumors. Data are presented as means ± SEM. Statistical significance was assessed using the Mann – Whitney *U* test, one-way ANOVA, Adonis, or an independent samples *t*-test, as appropriate. *p-*values: *p < 0.05; **p < 0.01; ***p < 0.001.

To test this hypothesis, we administered a broad-spectrum antibiotic solution (Abx) following chronic stress exposure in CAC mice to eliminate the resident gut microbiota (Figure 2(A)). Quantitative PCR analysis of the 16S rRNA gene revealed the depletion of total bacterial load following Abx treatment (Figure 2B), confirming the efficacy of microbiota depletion by Abx. Phenotypically, mice in the CAC+Stress+Abx group exhibited significantly fewer tumors, a lower overall tumor load, and fewer tumors exceeding 4 mm in diameter than those in the CAC+Stress group (Figure 2C). These findings indicate that the gut microbiota plays a crucial role in the growth of chronic stress-mediated CAC tumors.

To investigate the role of stress-altered gut microbiota in modulating tumor growth, we performed FMT using donor microbiota from either CAC or CAC+Stress mice (Figure 2D). Following transplantation, 16S rRNA gene sequencing revealed that the microbial communities of FMT recipients closely resembled those of their respective donors (Figure 2E). PCoA revealed tight clustering between the CAC+Stress+FMT-CAC and CAC groups, as well as between the CAC+Stress+FMT-Stress and CAC+Stress groups (Fig. S4B). Furthermore, PERMANOVA (Adonis) analysis confirmed the absence of any significant difference between the donor and FMT recipient groups (Figure 2F), indicating successful microbial engraftment. Mice receiving microbiota from CAC donors (CAC+Stress+FMT-CAC) had significantly fewer tumors, lower tumor burden, and fewer large-sized tumors than those receiving microbiota from stressed donors (CAC+Stress+FMT-Stress) (Figure 2G). Collectively, these findings suggest that chronic stress reshapes the composition and diversity of the gut microbiota, thereby accelerating the progression of CAC.

### *Chronic stress reduces the abundance of* A. muciniphila *in the gut and colorectal tumor tissues*

**Figure 3.**
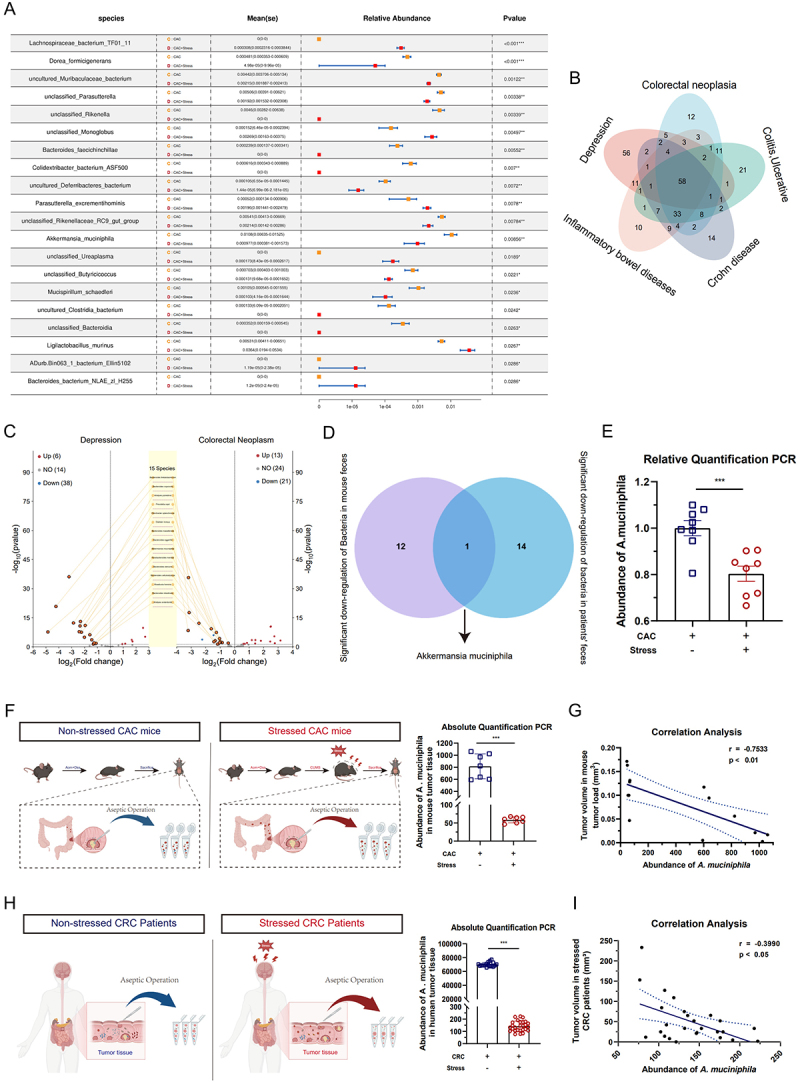
Chronic stress reduces the abundance of *akkermansia muciniphila* in the gut and colorectal tumor tissues. (A) Differences in the composition of gut microbial species between colitis-associated colorectal cancer (CAC) and CAC+Stress mice based on 16S rRNA sequencing (*n* = 6). (B) Shared bacterial species identified in patients with depression and colorectal cancer (CRC)-related conditions from the gut microbiota repository (GMrepo) database (*n* = 150). (C) Fifteen bacterial species are significantly downregulated in patients with depression and CRC among the 58 shared taxa. (D) Intersection of significantly downregulated bacteria in mice and patients. (E) Abundance of *A. muciniphila* in mouse feces (*n* = 8). (F and G) abundance of *A. muciniphila* in murine colorectal tumor tissues (*n* = 7) and its correlation with tumor volume (*n* = 14). (H and I) abundance of *A. muciniphila* in human colorectal tumor tissues (*n* = 65) and its correlation with tumor volume (*n* = 33). Data are presented as means ± SEM. Statistical significance was assessed using the Metastats permutation-based non-parametric test with false discovery rate (FDR) correction where appropriate, the Mann – Whitney *U* test, or an independent samples *t*-test, as appropriate. Correlation analyses were performed using Spearman’s rank correlation. *p-*values: ***p < 0.001.

To identify potential commensal bacteria consistently affected by chronic stress in the murine gut, we first conducted 16S rRNA gene sequencing on the intestinal contents of mice. Significant alterations in gut microbiota composition were observed between CAC and CAC+Stress mice at the species and genus levels (Figure 3A; Fig. S5A).

To explore commensal bacteria that were consistently altered by chronic stress in the human gut, we analyzed publicly available microbiome datasets including depression and CRC-related conditions. This strategy was based on two key considerations: (1) Depression is a well-established clinical manifestation of chronic psychological stress and widely used as a proxy in stress-related microbiome studies; (2) CRC frequently arises from chronic intestinal inflammation and microbial alterations observed in inflammatory bowel conditions may also be biologically relevant to CRC pathogenesis. Through this analysis, we identified 58 commensal bacterial species associated with both psychological stress and CRC (Figure 3B). Among them, 15 microbial species were significantly downregulated in both depression and CRC compared with healthy controls (Figure 3C; Fig. S5B).

By intersecting the human-derived downregulated species with those significantly reduced in stressed CAC mice, we showed that *A. muciniphila* was the only species consistently downregulated in the two datasets (Figure 3D). To confirm the sequencing results, we validated the abundance of *A. muciniphila* in mouse fecal samples using qPCR. The results confirmed that its abundance was significantly lower in the CAC+Stress group than in the CAC group (Figure 3E).

Nejman et al. demonstrated that the gut microbiota can colonize tumor tissues, thereby affecting tumor initiation and progression.^50^ Consequently, we investigated the abundance of *A. muciniphila* in the tumor tissues of stressed mice and patients. Consistent with the changes observed in the fecal microbiota, the abundance of *A. muciniphila* was significantly lower in the tumor tissues of stressed mice and patients than in their respective control groups (Figure 3F–H). Moreover, correlation analysis revealed a negative association between *A. muciniphila* abundance and tumor volume and number (Figure 3G–I; Fig. S5C and D).

### A. muciniphila suppresses the growth of chronic stress-mediated colorectal neoplasms

**Figure 4.**
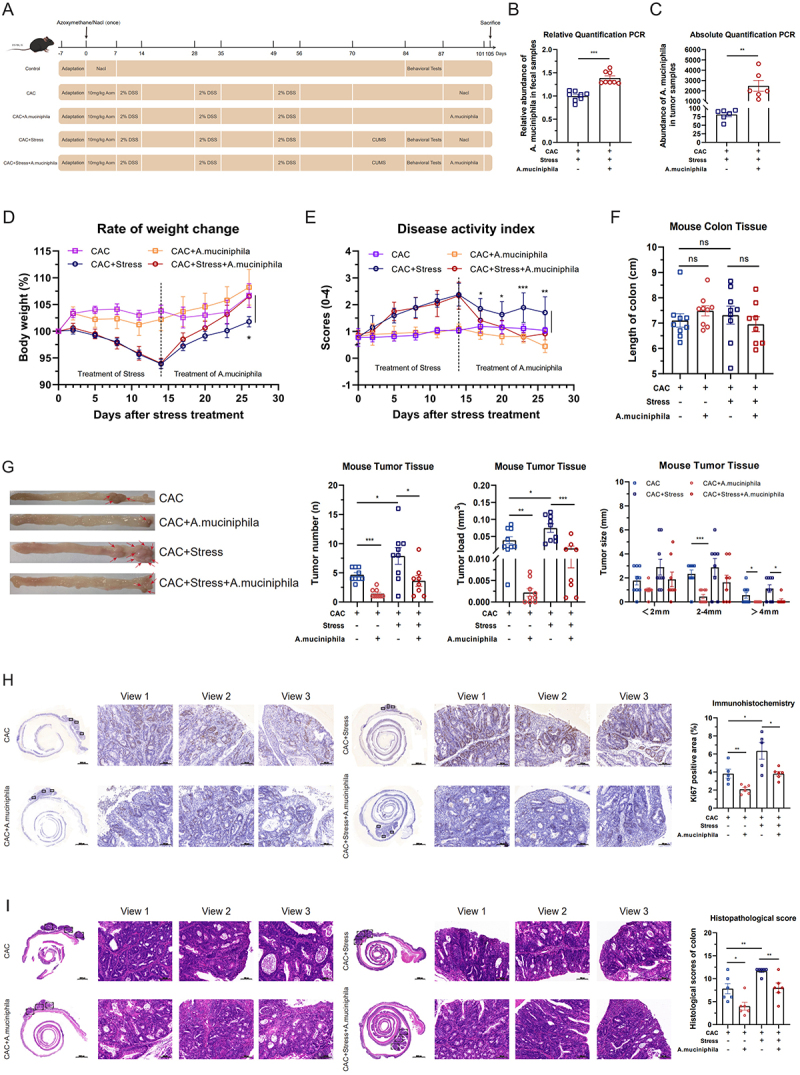
*Akkermansia muciniphila* suppresses the growth of chronic stress-mediated colorectal neoplasms. (A) Schematic diagram of the *A. muciniphila* gavage model. (B and C) abundance of *A. muciniphila* in fecal samples (*n* = 8) and tumor tissues (*n* = 6). (D–F) Effects of *A. muciniphila* gavage on the rate of body weight change, disease activity index, and intestinal length in mice (*n* = 9). (G) Quantitative analysis of tumor burden in chronic stress-induced colitis-associated colorectal cancer (CAC) mice supplemented with *A. muciniphila*. Tumor number, tumor load, and tumor size distribution were assessed (*n* = 6). Representative tumor images are shown to the left of the bar graphs, with red arrows indicating individual tumors. (H and I) effects of *A. muciniphila* gavage on Ki67 expression and histopathological changes in tumor tissues of stressed mice (*n* = 5–6). Scale bar = 100 μm. Data are presented as means ± SEM. Statistical significance was assessed using the Mann – Whitney *U* test, one-way ANOVA, or an independent samples *t*-test, as appropriate. *p-*values: ^ns^ *p* > 0.05; *p < 0.05; **p < 0.01; ***p < 0.001.

We investigated the potential role of *A. muciniphila* in the growth of chronic stress-mediated CAC tumors. To replenish *A. muciniphila*, we administered a 14-day oral gavage of *A. muciniphila* to chronic stress-induced CAC mice (Figure 4A). A significant increase in *A. muciniphila* abundance was observed in the feces and tumor tissues of mice in the gavage group compared with that in the control group (Figure 4B,C), indicating the successful supplementation of *A. muciniphila* in the intestine of stressed mice.

During this period, we assessed the effects of *A. muciniphila* on changes in body weight, DAI score, and intestinal tissue length. Compared with that in the CAC+Stress group, *A. muciniphila* supplementation alleviated stress-induced body weight loss and elevated DAI (Figure 4D,E). However, no significant changes in the intestinal tissue length were observed (Figure 4F). To evaluate the impact of *A. muciniphila* on the progression of chronic stress-induced CAC, we performed a gross examination of the colonic tissue, IHC, and tumor histopathological scoring. Gross examination revealed that *A. muciniphila* supplementation significantly mitigated the promotion of tumor growth induced by chronic stress, as evidenced by a reduction in the total tumor number, tumor load, and number of tumors exceeding 4 mm in diameter (Figure 4G). IHC revealed that *A. muciniphila* supplementation suppressed stress-induced cell proliferation in CAC tumor tissues, as indicated by a significant reduction in the number of Ki67-positive proliferating cells (Figure 4H). Furthermore, *A. muciniphila* supplementation effectively attenuated the stress-induced malignant progression in CAC mice, as reflected by a significantly reduced histopathological score of tumor tissues (Figure 4I).

### A. muciniphila OMVs suppress the growth of chronic stress-induced colorectal neoplasms

**Figure 5.**
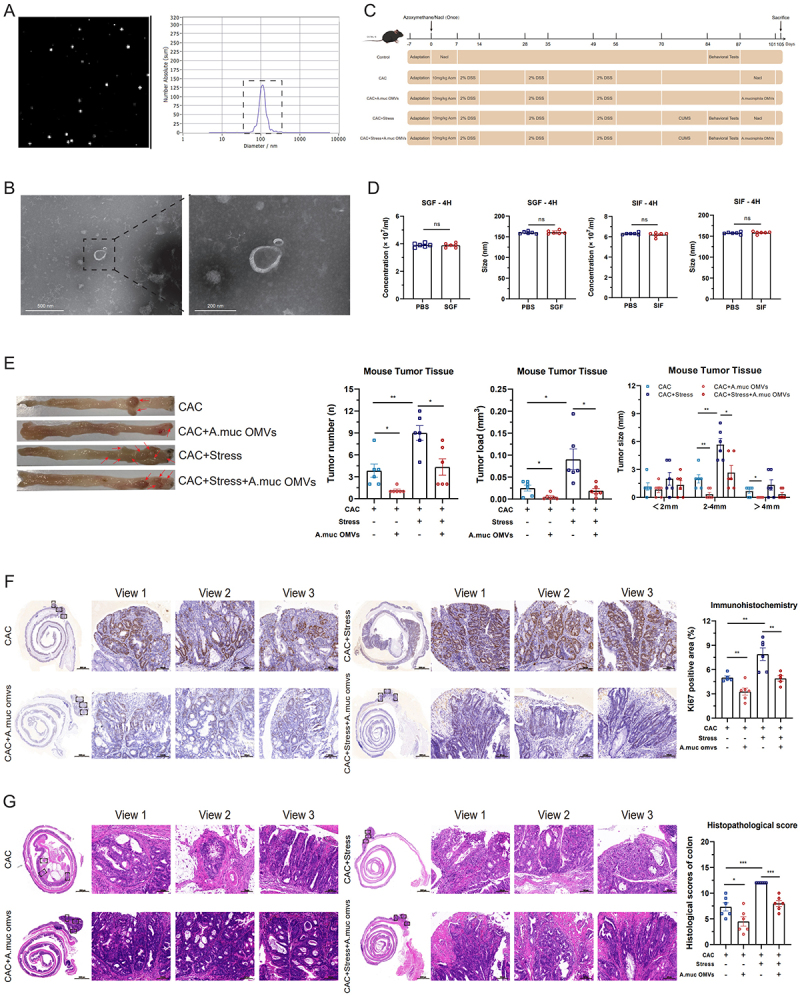
*Akkermansia muciniphila* outer membrane vesicles (OMVs) suppress the growth of chronic stress-induced colorectal neoplasms. (A and B) Characterization of purified *A. muciniphila* OMVs via nanoparticle tracking analysis and transmission electron microscopy. (C) Schematic diagram of the *A. muciniphila* OMV gavage model. (D) Stability of *A. muciniphila* OMVs after 4 h of incubation in simulated gastric fluid (SGF) and simulated intestinal fluid (SIF). (E) quantitative analysis of tumor burden in chronic stress-induced colitis-associated colorectal cancer (CAC) mice supplemented with *A. muciniphila* OMVs. Tumor number, tumor load, and tumor size distribution were assessed (*n* = 6). Representative tumor images are shown to the left of the bar graphs, with red arrows indicating individual tumors. (F and G) Effects of *A. muciniphila* OMVs gavage on Ki67 expression and histopathological changes in tumor tissues of stressed mice (*n* = 5–6). Scale bar = 100 μm. Data are presented as means ± SEM. Statistical significance was assessed using one-way ANOVA or an independent samples *t*-test, as appropriate. *p-*values: ^ns^ *p* > 0.05; *p < 0.05; **p < 0.01; ***p < 0.001.

Building upon the abovementioned findings, we investigated how *A. muciniphila* suppresses tumor growth in chronic stress-induced CAC. We cultured intestinal tumor cell lines in the basal culture medium of supernatant obtained from *A. muciniphila* to examine the effects of its metabolites on the proliferation of intestinal tumor cells. Compared with the basal culture medium, the *A. muciniphila* supernatant significantly inhibited the proliferative activity of intestinal tumor cell lines (Fig. S6A). This suggests that the *A. muciniphila* supernatant contains unidentified components capable of suppressing the intestinal tumor cell activity. During its growth, *A. muciniphila* naturally releases specialized derivatives containing parental proteins, nucleic acids, enzymes, and lipopolysaccharides. These derivatives constitute a highly conserved and sophisticated bacterial – host communication system.^53^ *A. muciniphila* secretes OMVs, which contribute to the enhancement of intestinal barrier function.^38^ We, therefore, hypothesized that *A. muciniphila* OMVs play a pivotal role in inhibiting stress-related CAC progression. The supernatant was collected after bacterial culture and purified via ultracentrifugation. NTA and TEM revealed that the extracted vesicles had an average diameter of 110.60 ± 60.3 nm and exhibited the characteristic cup-shaped and spherical bilayered nanostructures (Figure 5A,B), consistent with previously reported descriptions of *A. muciniphila* OMVs.^54^

To investigate the effects of *A. muciniphila* OMVs on tumor progression in chronic stress-induced CAC mice, we orally administered OMVs for 14 days (Figure 5C). Prior to administration, the OMVs were incubated in SGF and SIF at 37°C for 2 and 4 h, respectively. No significant changes in their concentration or size were observed, which indicated that *A. muciniphila* OMVs would exhibit satisfactory stability when passed through the digestive system (Figure 5D; Fig. S6B). During the oral administration of *A. muciniphila* OMVs, we monitored the rate of body weight change and DAI in each group of mice. Compared with the CAC+Stress group, the oral administration of *A. muciniphila* OMVs alleviated stress-induced weight loss and increased DAI (Fig. S6C and D). To evaluate the effect of *A. muciniphila* OMVs on the progression of chronic stress-mediated CAC, we performed a macroscopic examination of the colonic tissue, IHC, and tumor histopathological scoring. Macroscopic analysis revealed that, compared with CAC+Stress mice, the oral administration of *A. muciniphila* OMVs significantly reduced the total tumor number, tumor load, and number of tumors measuring 2–4 mm in diameter (Figure 5E). IHC analysis showed that the administration of *A. muciniphila* OMVs attenuated the pro-proliferative effects of chronic stress on colorectal tumors, as evidenced by a significant reduction in Ki67-positive proliferating cells (Figure 5F). Moreover, *A. muciniphila* OMVs significantly mitigated the malignant progression of CAC under chronic stress, as reflected by a substantial decrease in histopathological scores (Figure 5G).

### A. muciniphila OMVs suppress the proliferation of CRC cell lines

**Figure 6.**
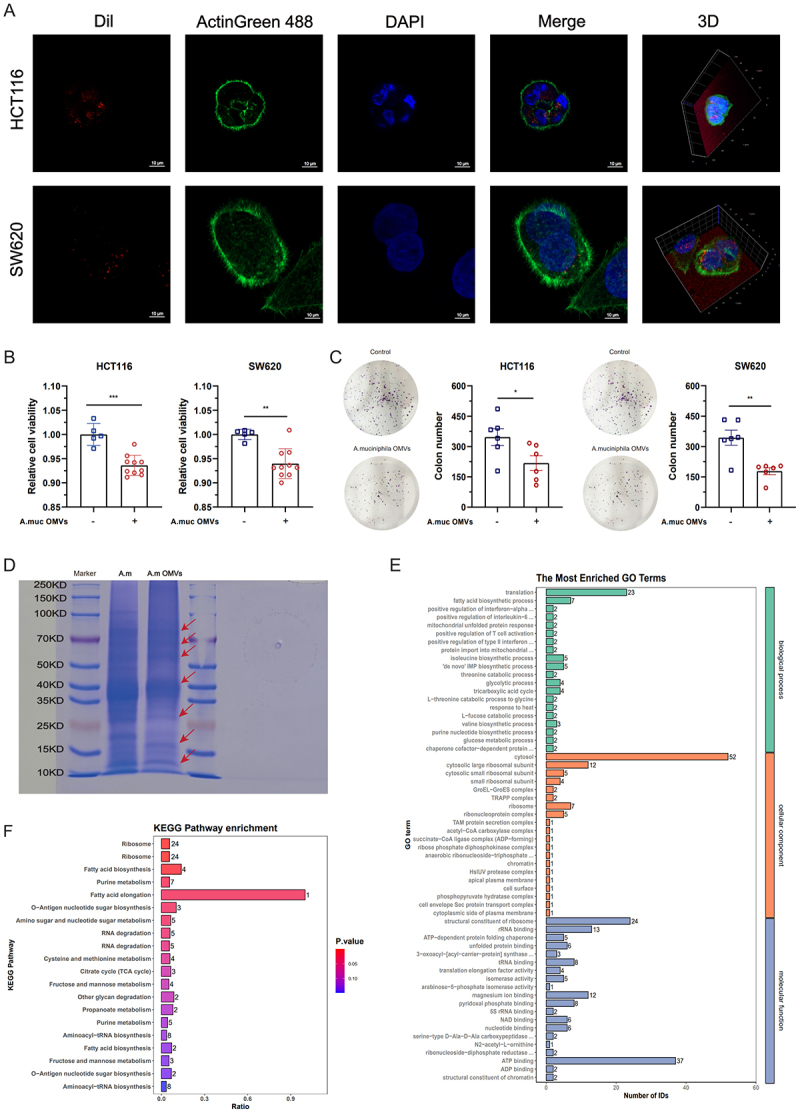
*Akkermansia muciniphila* outer membrane vesicles (OMVs) suppress the proliferation of colorectal cancer (CRC) cell lines. (A) Uptake of *A. muciniphila* OMVs by CRC cell lines. Representative confocal fluorescence images showing the uptake of Dil-labeled OMVs (red) by CRC cell lines HCT116 and SW620. Actin filaments were stained with ActinGreen 488 (green), and nuclei were counterstained with DAPI (blue). Merged images indicate intracellular localization of OMVs relative to the cytoskeleton and nucleus. Scale bar = 10 μm. (B and C) Effects of *A. muciniphila* OMVs on CRC cell proliferation and colony formation ability (*n* = 5–10). (D) Coomassie brilliant blue staining of total proteins extracted from *A. muciniphila* and its OMVs. Red arrows indicate protein bands selectively enriched in OMVs. (E and F) Gene ontology (GO) and Kyoto encyclopedia of genes and genomes (KEGG) analysis of proteins enriched in *A. muciniphila* OMVs. Data are presented as means ± SEM. Statistical significance was assessed using an independent samples *t*-test. *p-*values: * *p* < 0.05; ** *p* < 0.01; *** *p* < 0.001.

To investigate the effects of *A. muciniphila* OMVs on CRC cell proliferation, we examined the uptake of *A. muciniphila* OMVs by CRC cell lines. CRC cells readily internalized *A. muciniphila* OMVs (Figure 6A, Fig. S7A), suggesting a fundamental interaction between *A. muciniphila* OMVs and CRC cells. Upon coculture of CRC cells with *A. muciniphila* OMVs, *A. muciniphila* OMVs significantly attenuated cell viability and colony formation capacity (Figure 6B,C). To evaluate the specificity of *A. muciniphila* OMVs to tumor cells, we assessed their effect on CRC cell lines and normal human colonic epithelial cells. Although OMVs reduced CRC cell viability, they exerted no effect on NCM460 cells (Fig. S7B), indicating their selective antitumor activity with minimal cytotoxicity toward normal cells.

To characterize *A. muciniphila* OMVs, we performed CBB staining to assess protein enrichment. During OMV secretion, *A. muciniphila* selectively and actively enriched certain parental proteins (Figure 6D). Subsequently, we analyzed the composition and functional attributes of the proteins enriched in *A. muciniphila* OMVs. In total, 226 proteins were stably enriched in *A. muciniphila* OMVs (Fig. S7C). Gene ontology (GO) analysis revealed that these proteins are involved in 20 biological processes, 20 cellular components, and 20 molecular functions (Figure 6E). Kyoto Encyclopedia of Genes and Genomes (KEGG) pathway analysis indicated that these proteins primarily participate in fatty acid biosynthesis and elongation (Figure 6F).

## Discussion

**Figure 7.**
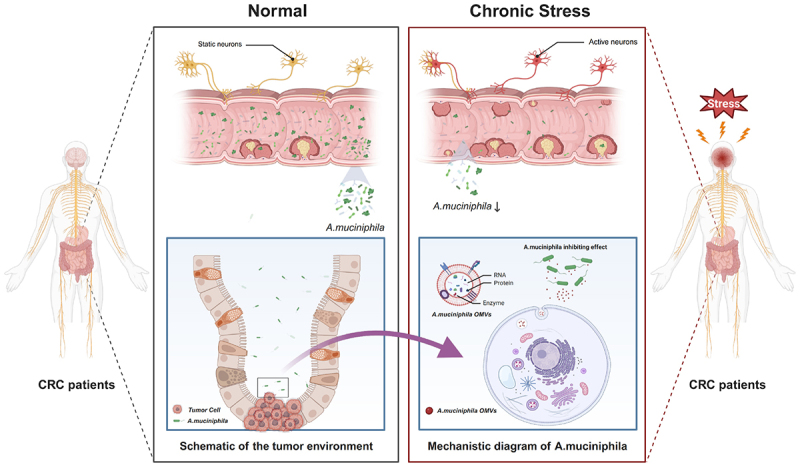
Chronic stress promotes the progression of colorectal cancer (CRC) by reducing the abundance of the probiotic *akkermansia muciniphila*, impairing its outer membrane vesicle (OMV)-mediated antitumor effects.

In the present study, we confirmed that chronic stress is a significant risk factor for the progression of colorectal neoplasm and found that the gut microbiota plays a critical role in this process. Subsequently, we observed that stress reduced the abundance of *A. muciniphila* in the gut and tumor tissues of patients with CRC and CAC mice, whereas restoring the abundance of this bacterium significantly mitigated the tumor-promoting effects of chronic stress. Furthermore, we identified *A. muciniphila* OMVs as key mediators of these antitumor effects (Figure 7). A recent study by Chui et al. supports our hypothesis that chronic stress promotes tumor progression through gut microbiota dysbiosis and is particularly characterized by the depletion of *A. muciniphila*.^55^ Although their study focused on breast cancer, their findings are highly consistent with our observations in colorectal neoplasm, suggesting that the stress – gut – tumor axis represents a conserved mechanism across different cancer types.

*A. muciniphila*, a mucinophilic, gram-negative anaerobic bacterium native to the mammalian intestinal ecosystem, primarily colonizes the gut mucus layer and relies on mucin as its principal nutrient source.^56–58^ A recent study by Allen et al. demonstrated that chronic stress activates oxidative stress pathways and inflammatory signaling within intestinal epithelial cells, resulting in disruption to the mucosal barrier, as evidenced by the thinning of the mucus layer, increased intestinal permeability, and microbial translocation.^59^ Based on these findings, we hypothesize that stress-induced perturbations of the mucosal environment may compromise the ecological niche of *A. muciniphila*, thereby contributing to its significant depletion. In future studies, we plan to validate this hypothesis by developing models of mucus layer disruption and implementing barrier repair strategies to elucidate the mechanistic relevance of this pathway in stress-related tumor progression.

Our findings provide compelling evidence that *A. muciniphila*-derived OMVs exert antitumor effects against colorectal neoplasm in vitro and in vivo. We observed that *A. muciniphila* selectively packages specific proteins into OMVs during secretion, with a total of 226 proteins identified as consistently enriched in the OMVs. This observation suggests that these actively enriched components play a critical role in mediating the antitumor activity of this bacterium. Consistent with this notion, Wang et al. identified a unique outer membrane protein from *A. muciniphila*, Amuc_1100, which was shown to inhibit the development of CAC.^40^ Moreover, in our previous study, we observed that the oral administration of *A. muciniphila* or its OMVs significantly alleviates stress-related behavioral symptoms. This suggests that the observed antitumor effects are partially mediated by a reduction in chronic stress, in addition to direct cytotoxic effects on tumor cells.^60^ Furthermore, a recent study by Fan et al. demonstrated that *A. muciniphila* suppresses CRC progression by activating the TLR2/NLRP3 signaling pathway and promoting the M1-like polarization of tumor-associated macrophages.^37^ These findings suggest that, in addition to the effects mediated by OMV release, *A. muciniphila* modulates the tumor immune microenvironment to inhibit tumor growth. In summary, the use of *A. muciniphila* may constitute a multifaceted antitumor strategy involving OMV-mediated molecular signaling and the modulation of host immune and neuroendocrine pathways.

Although our experiments demonstrated that the oral administration of *A. muciniphila* effectively suppresses the progression of colorectal neoplasms under chronic stress conditions and clinical studies have confirmed its safety in humans,^61^ its application as an antitumor therapy still faces several practical challenges. As a strict anaerobe, *A. muciniphila* is highly sensitive to oxygen, temperature, and pH, which may compromise its viability, bioactivity, and colonization efficiency under suboptimal conditions.^62^ Given the potent antitumor effects observed with *A. muciniphila*-derived OMVs, along with their superior safety profile, stability, and ease of administration to live bacteria, OMV-based biotherapeutics represent a promising strategy for controlling tumor progression in patients with CRC experiencing chronic psychological stress. This study not only elucidates a novel microbiota-derived mechanism underlying stress-aggravated CRC progression but also provides a conceptual framework for OMV-based therapeutic development.

Owing to limitations in the availability of clinical patient data, this study included only sex, age, and stress status as potential clinical variables associated with tumor growth. Several important potential confounding factors – such as diabetes, obesity, smoking status, alcohol consumption, physical activity, and dietary habits – were not included in the analysis. In future studies, we plan to use larger sample sizes and incorporate more comprehensive clinical datasets to clarify the independent role of chronic stress in CRC progression and elucidate its interaction with lifestyle-related risk factors.

## Supplementary Material

02._Revised_supplementary_materials__1.docx

## Data and materials availability statement

Data needed to evaluate the conclusions in the paper are present in the paper and/or the Supplementary Materials. The data have been deposited in the Mendeley Data database (10.17632/nsf8877y48.1).
