# Supplementary material for: Akkermansia muciniphila ameliorates chronic stress-induced colorectal tumor growth by releasing outer membrane vesicles
Source: Gut Microbes. 2025 Sep 8;17(1):2555618. doi: 10.1080/19490976.2025.2555618 (PMC12427454; doi:10.1080/19490976.2025.2555618)
Supplement: 02._Revised_supplementary_materials__1.docx [file KGMI_A_2555618_SM5086.docx]

Supplementary Materials for

***Akkermansia muciniphila* ameliorates chronic stress-induced colorectal tumor growth by releasing outer membrane vesicles**

Shunkang Jin and Yanjie Lu *et al*.

*Corresponding author. Email: Yuhong Li, lyhky@cdmc.edu.cn; Shuai Wang, shuai_shuai2006@163.com

**This PDF file includes:**

Figs. S1 to S7


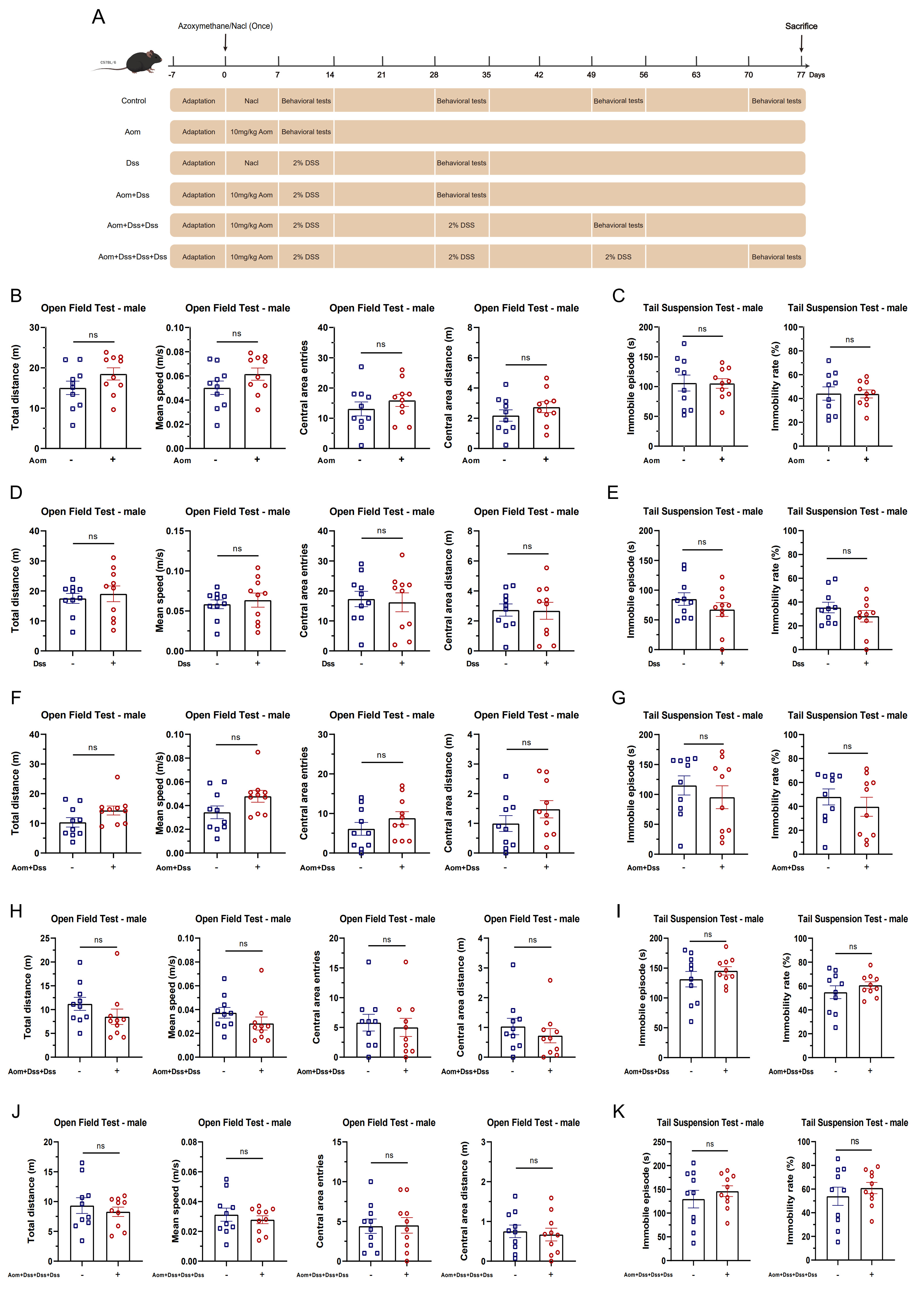
**Fig. S1** Chronic pain induced during colitis-associated colorectal cancer (CAC) modeling does not trigger significant chronic stress in male mice. (**A**) Overview of the experimental design. Behavioral performance of male mice in open field test (OFT) and tail suspension test (TST) following (**B** and **C**) azoxymethane (AOM), (**D** and **E**) dextran sulfate sodium (DSS), (**F** and **G**) AOM+DSS, (**H** and **I**) AOM+DSS+DSS, and (**J** and **K**) AOM+DSS+DSS+DSS treatments (*n* = 10 for each). Data are presented as means ± SEM. Statistical significance was assessed using an independent samples t-test. *P-*values: ^ns^ *P* > 0.05.

**
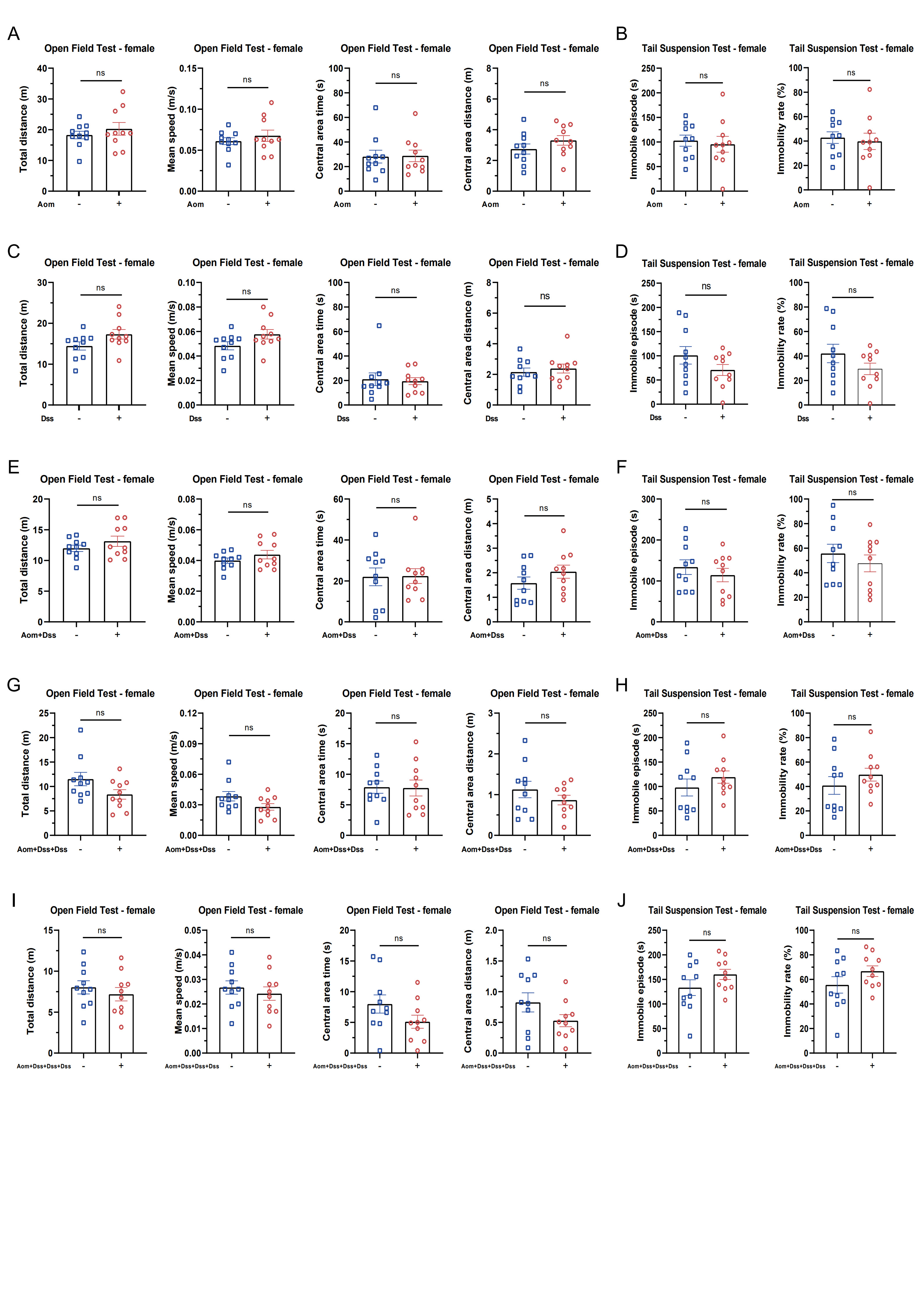
Fig. S2** Chronic pain induced during colitis-associated colorectal cancer (CAC) modeling does not trigger significant chronic stress in female mice. Behavioral performance of female mice in open field test (OFT) and tail suspension test (TST) following (**A** and **B**) azoxymethane (AOM), (**C** and **D**) dextran sulfate sodium (DSS), (**E** and **F**) AOM+DSS, (**G** and **H**) AOM+DSS+DSS, and (**I** and **J**) AOM+DSS+DSS+DSS treatments (*n* = 10 for each). Data are presented as means ± SEM. Statistical significance was assessed using an independent samples t-test. *P-*values: ^ns^ *P* > 0.05.


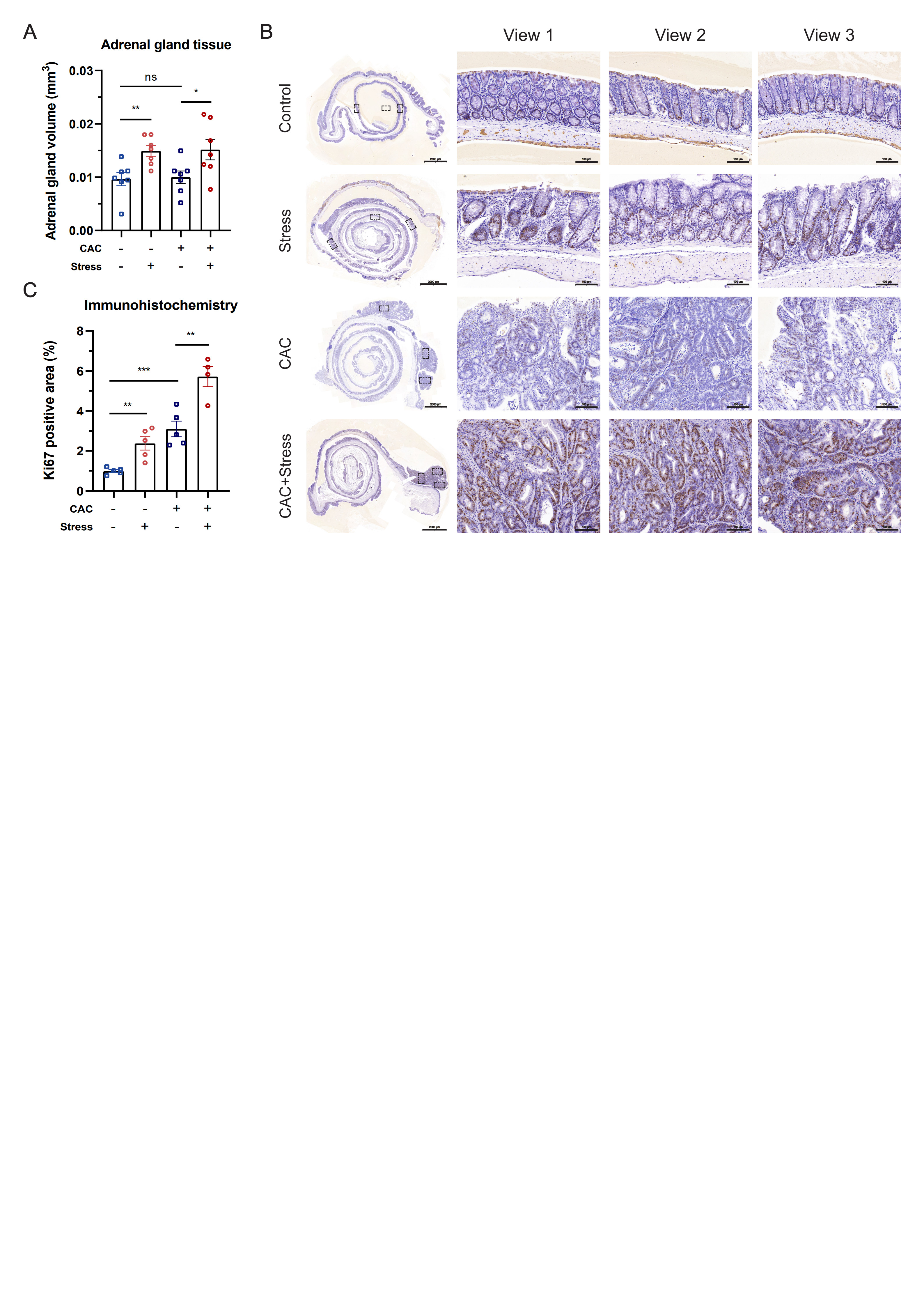


**Fig. S3** Chronic stress promotes tumor cell proliferation. Effect of chronic stress on (**A**) adrenal gland volume (*n* = 7) and (**B** and **C**) Ki67 expression in tumor tissues (*n* = 5–6). Scale bar = 100 μm. Data are presented as means ± SEM. Statistical significance was assessed using one-way ANOVA. *P-*values: ^ns^ *P* > 0.05; ^*^ *P* < 0.05; ^**^ *P* < 0.01; ^***^ *P* < 0.001.


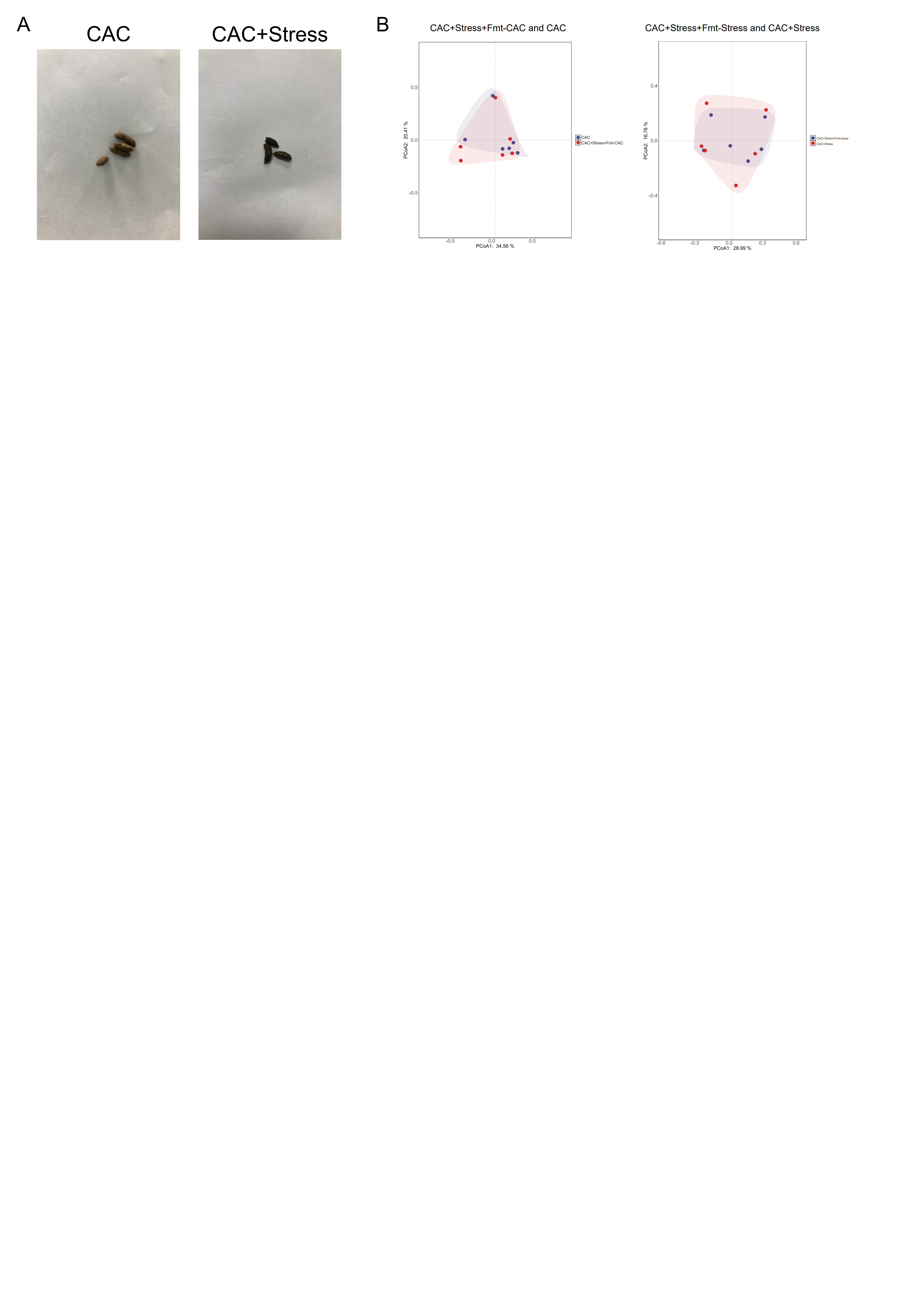


**Fig. S4** Chronic stress alters fecal characteristics. (**A**) Changes in mouse fecal characteristics following chronic stress. (**B**) Principal coordinate analysis (PCoA) of β-diversity was performed on fecal samples from donor and recipient mice (*n* = 6).


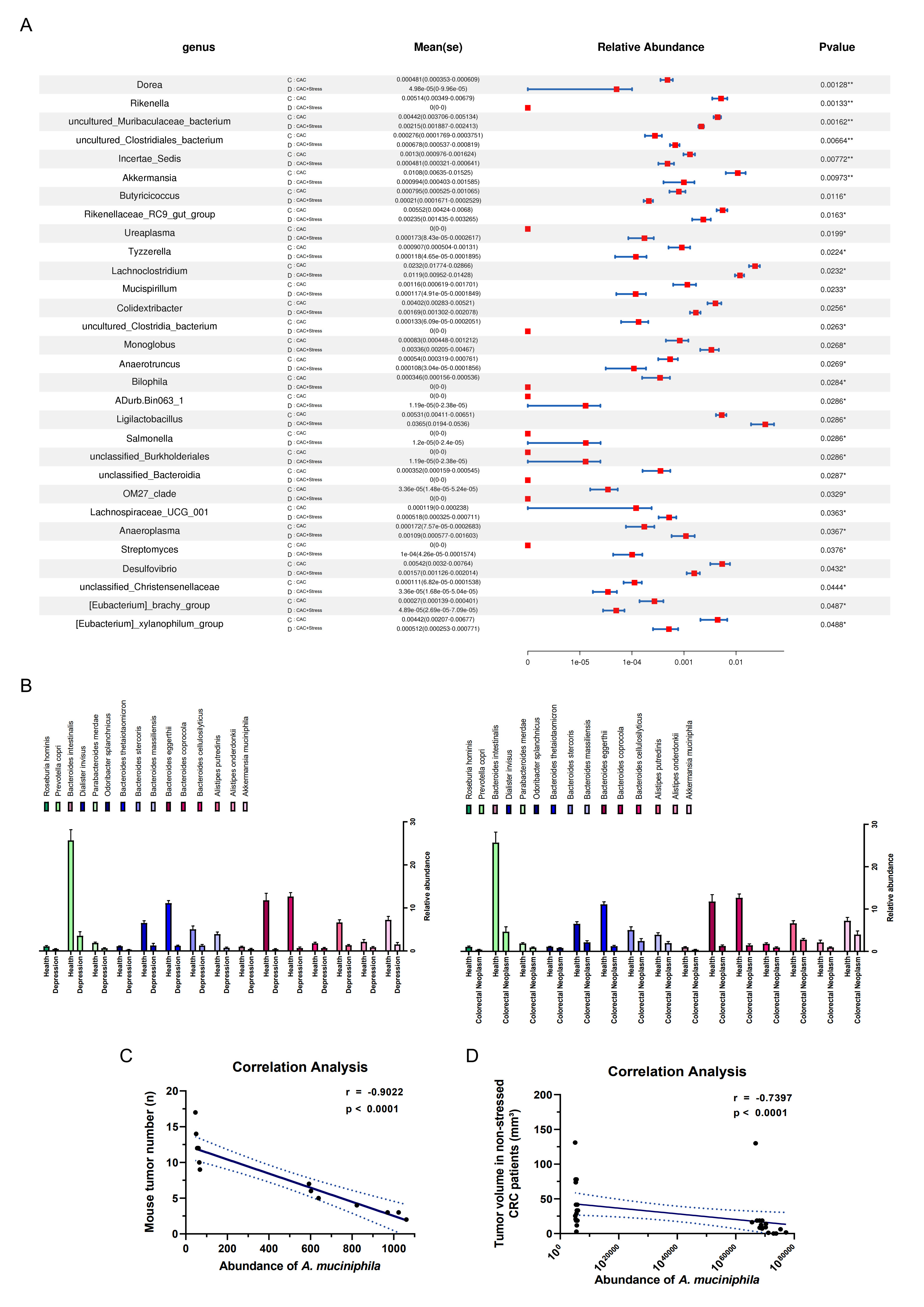


**Fig. S5** Stress-associated commensal bacteria commonly downregulated in both mice and humans. (**A**) Differences in gut microbiota composition at the genus level between colitis-associated colorectal cancer (CAC) and CAC+Stress mice based on 16S rRNA sequencing (*n* = 6). (**B**) Significantly downregulated bacterial species in patients with depression or colorectal cancer (CRC) (*n* = 58). (**C** and **D**) Correlation analysis between *Akkermansia muciniphila* abundance and tumor number or volume in the tumor tissues of CAC mice (*n* = 14) and patients with CRC (*n* = 32). Data are presented as means ± SEM. Statistical significance was assessed using the Metastats, a permutation-based non-parametric method tailored for microbiome data, with FDR correction applied for multiple comparisons where appropriate.


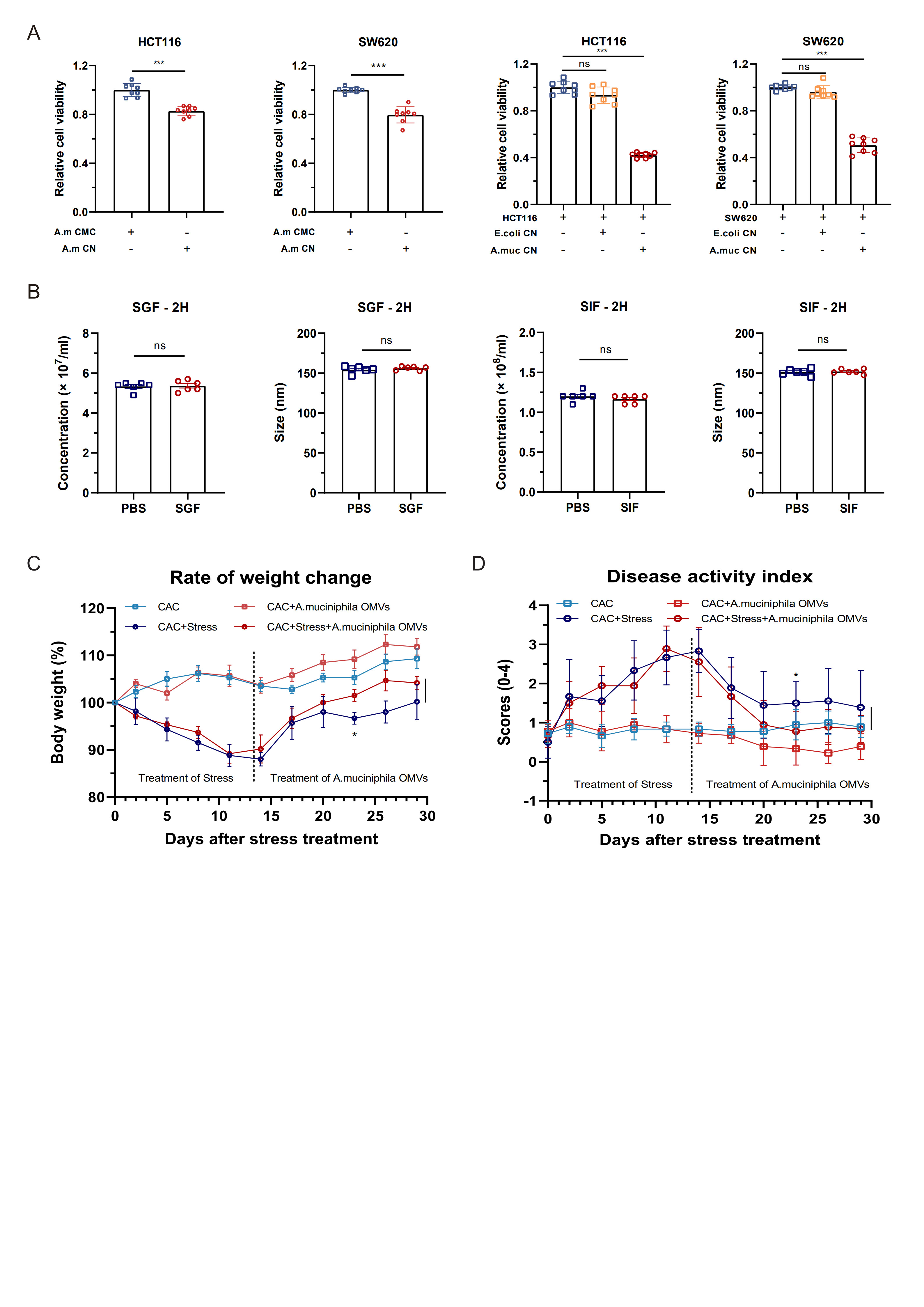


**Fig. S6** Effect of *Akkermansia muciniphila* metabolites on the proliferative activity of colorectal cancer (CRC) cells. (**A**) Effect of *A. muciniphila* supernatant on the proliferative activity of HCT116 and SW620 cells (*n* = 8). (**B**) Stability of *A. muciniphila* outer membrane vesicles (OMVs) after 2 h of incubation in simulated gastric fluid (SGF) and simulated intestinal fluid (SIF). (**C** and **D**) Effects of *A. muciniphila* OMVs gavage on the rate of body weight change and DAI in mice (*n* = 6). Data are presented as means ± SEM. Statistical significance was assessed using an independent samples t-test. *P-*values: ^ns^ *P* > 0.05; ^*^ *P* < 0.05; ^***^ *P* < 0.001.


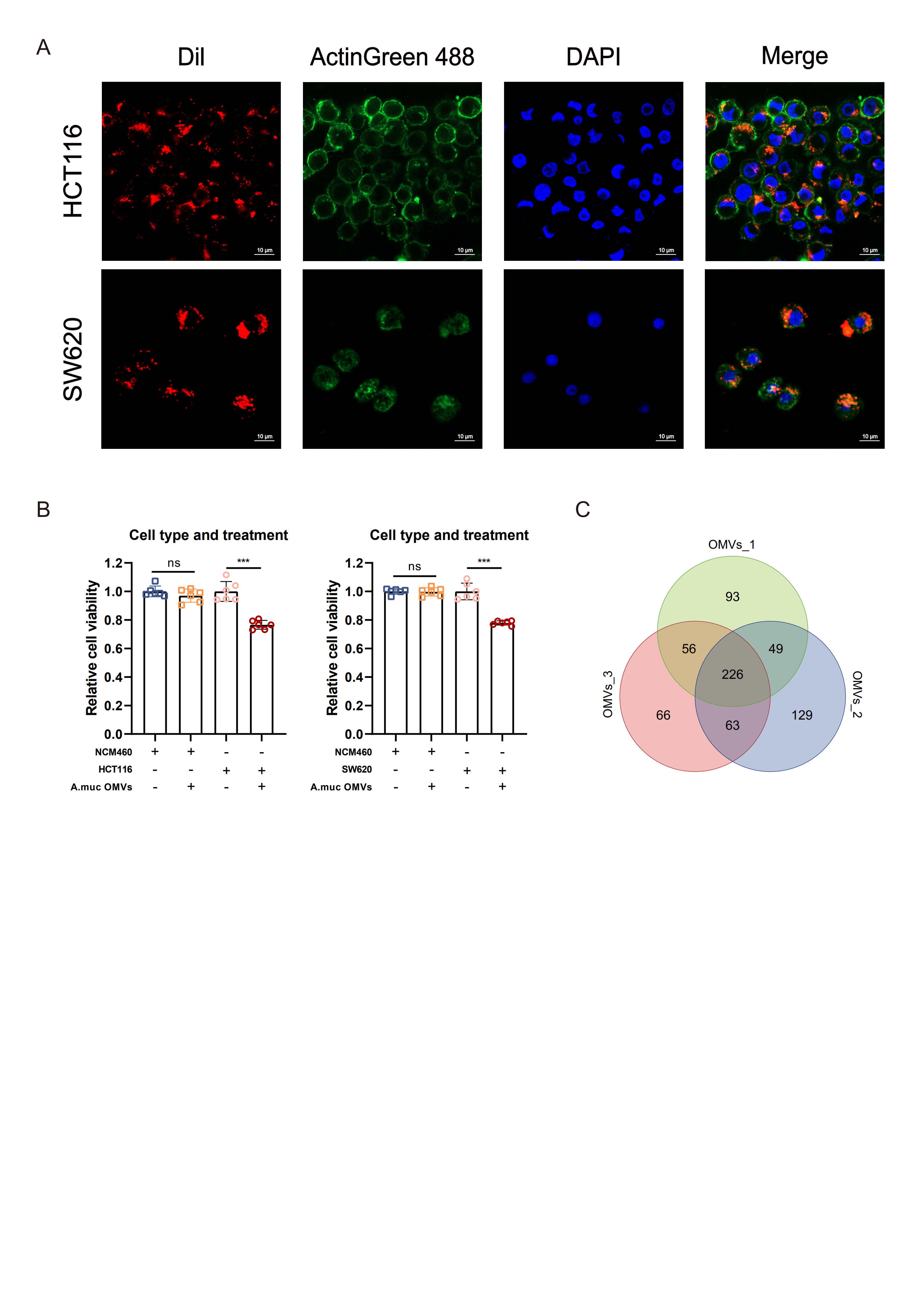


**Fig. S7** Cellular uptake and protein composition of *Akkermansia muciniphila*-derived outer membrane vesicles (OMVs). **(A)** Representative confocal fluorescence images show the uptake of Dil-labeled OMVs (red) by colorectal cancer (CRC) cell lines HCT116 and SW620. Actin filaments were stained with ActinGreen 488 (green) and nuclei were counterstained with DAPI (blue). Merged images indicate intracellular localization of OMVs relative to the cytoskeleton and nucleus. Scale bar = 10 μm. **(B)** Effects of *A. muciniphila* OMVs on cell viability in normal colon epithelial cells (NCM460) and CRC cell lines (HCT116 and SW620). **(C)** Venn diagram illustrating the overlap of proteins identified in three independently prepared OMV samples (OMVs_1, OMVs_2, OMVs_3) using LC-MS/MS analysis. In total, 226 proteins were consistently detected across all three replicates, indicating the stable enrichment of core protein components within OMVs. Data are presented as means ± SEM. Statistical significance was assessed using one-way ANOVA. *P-*values: ^ns^ *P* > 0.05; ^***^ *P* < 0.001.
